# Two-Stage Multi-View Graph Spectral Clustering for Single-Cell RNA-Seq Data

**DOI:** 10.2174/0113892029387405250921211549

**Published:** 2025-10-10

**Authors:** Lianlian Zhang, Junliang Shang, Xiangzhen Kong, Feng Li, Jin-Xing Liu

**Affiliations:** 1 School of Computer Science, Qufu Normal University, Rizhao, 276826, China;; 2 School of Health and Life Sciences, University of Health and Rehabilitation Science, Qingdao, 266113, China

**Keywords:** scRNA-seq, multi-view graph, spectral clustering, multi-view feature and structure network, clustering algorithm, clustering analysis

## Abstract

**Introduction:**

The appearance of single-cell RNA sequencing (scRNA-seq) data has brought a distinctive perspective to studying gene expression at the cell level. However, it faces challenges such as large data volume, sparsity, heterogeneity, and the curse of dimensionality. Current clustering methods still face many challenges in studying cell type distribution and have not utilized the structural relationship information between cells.

**Methods:**

To avoid the insufficiency of the single characteristic space of scRNA-seq data in characterizing cell function, this paper constructs multiple view characteristic spaces and utilizes multi-view learning to characterize scRNA-seq information from distinctive perspectives comprehensively. In multi-view learning, the similarity graph is divided into weighted learning and structural learning stages. Through weighting the multi-view similarity graphs, the significance of diverse views and features is underscored. During the structural stage, the emphasis is placed on uncovering potential relationships among different views by preserving common edges in the multi-view similarity graphs. The optimization of the attribute and structure graphs was conducted separately by the alternating direction multiplier method.

**Results:**

The performance of the MVGSC was validated using eight different scales of real scRNA-seq datasets, and the experimental results showed that the proposed multi-view clustering method significantly surpasses other single-view clustering methods and multi-view clustering methods.

**Discussion:**

When the features of scRNA-seq data are complex and there are significant differences between views, the two-stage multi-view graph method can better capture the complex relationships in the data, demonstrating superior performance compared to a single framework.

**Conclusion:**

Two-stage multi-view learning can more accurately capture complex relationships in the data, thereby improving the accuracy of the model. It can also better capture consistency and complementary information in multi-view data, thereby enhancing the generalization ability of the model.

## 1 INTRODUCTION

The scRNA-seq technology can reveal differences between different cell types. The research on cell development, diseases, and treatments is of great significance. Cell clustering is a central task in the analysis of scRNA-seq data, involving the grouping of cells based on similarity in gene expression in individual cells. The same kind of cells are grouped into one class, and different kinds of cells are grouped into different clusters, thereby revealing the heterogeneity of cell populations [1-3].

Many studies focus on identifying cell types using scRNA-seq data, a process known as cell type clustering. The clustering algorithm is a key issue in cell clustering [4]. K-means [5] partitions data samples into k mutually exclusive clusters by assigning cells to the nearest cluster based on a distance function. However, k-means is very sensitive to initial cluster centers, which might cause the algorithm to converge to local optima, resulting in unstable clustering outcomes. SinNLRR [6] imposes non-negative low-rank structures on the similarity matrix and simultaneously introduces an adaptive penalty selection approach to reduce the sensitivity of model parameters. As clustering algorithms evolve, graph-based methods are increasingly used to distinguish between nonlinear mixtures of cell types by investigating the topological relationships among cells. SIMLR [7] introduces single-cell interpretation through multi-kernel learning, derives similarity measures from scRNA-seq data, and improves clustering results. Spectral clustering [8] treats all data points as connected by edges in space, introducing the concept of degrees, and employs k-means for clustering after these steps.

The single-cell clustering methods mentioned above belong to single-view clustering, but research has shown that single-view clustering solely relies on information from a single view, which fails to reveal the full complexity of the data. On the other hand, multi-view clustering combines information from different views or feature spaces, capturing various aspects or characteristics of the data. Different views typically contain complementary information that may be overlooked in single-view clustering. Multi-view clustering exploits this complementary information to offer deeper data insights. Additionally, when one view is affected by noise or outliers, information from other views can still contribute to clustering, enhancing the algorithm's robustness.

Multi-view clustering methods are generally divided into three types: collaborative clustering, subspace clustering, and multi-kernel clustering. These methods are designed to maximize the similitude among distinctive views. Collaborative clustering was initially used in supervised multi-view learning and later evolved into unsupervised methods, such as multi-view spectral clustering and co-regularized multi-view spectral clustering. Some methods still employ unsupervised collaborative learning strategies to utilize auxiliary information. Multi-view collaborative clustering and subspace clustering methods are used to learn latent representation subspaces of data, contributing to multi-view clustering. Multi-kernel clustering is employed to handle multi-view features by integrating features through constructing kernels between different views. These methods typically require applying spectral clustering or k-means on the learned graph to get clustering results. Multi-view clustering is usually divided into two steps: first constructing graphs or kernels, and then obtaining clustering labels through learning algorithms. This two-step approach has a drawback in that the final clustering results cannot explicitly use a learned graph for representation, requiring another iteration of the k-means operation to obtain clustering indicators. GMC [9] obtains multiple graphs from all views and integrates them to produce a uniform graph. COMIC [10] achieves a certain degree of geometric consistency and cluster-matching consistency by spatially mapping the raw data. MCLES [11] performs clustering on multi-view data and learns the overall structure and cluster index matrix through a unified optimization framework.

Multi-similarity graph learning enriches the representation and feature learning of graph data by utilizing different similarity measures, enabling the understanding of complex relationships between nodes in graph data. Different similarity measures can capture associations at different levels between nodes, thus offering a more integrated understanding of the structure and features of graph data. Multiple Kernel Learning (MKL) learns the representation of data by linearly or nonlinearly combining multiple kernel functions. Each kernel function can be regarded as a similarity measure, and the combination of different kernel functions enriches the data representation and effectively integrates information from distinct perspectives, enhancing the learning and analysis capabilities of graph data representation. SIMLR [7] applies the MKL framework, enabling the learning of inter-cellular similarity measurements from scRNA-seq data. SMSC [12] utilizes the similarity information automatically learned from the data to transform candidate solutions into new solutions closer to discrete solutions, enabling the direct discovery of groups for scRNA-seq data.

Existing unsupervised multi-view feature selection methods largely have the following issues: the similarity matrix of samples, the weight matrix of different views, and the weight matrix of features are often predefined, failing to effectively capture the true structure of the data and reflect the importance of distinct views and features, consequently resulting in the inability to select valuable features. To address the aforementioned issues, AWMVPL [13], considering the correlation between views, learns the weights of distinct views through a self-weighting scheme. Moreover, by utilizing adaptive weighting, it integrates the information learned from view-specific neighborhood matrices into the matrix of common clustering indicators, which ultimately outputs the final clustering results. ALMUFS [14], building upon multi-view clustering, conducts adaptive learning of view weights and feature weights. This approach facilitates simultaneous feature selection while ensuring clustering performance. Moreover, under Laplacian rank constraints, it adaptively learns the similarity matrix of samples and constructs a multi-view unsupervised feature selection method based on adaptive learning.

In multi-view clustering, due to data coming from multiple different views or feature spaces, these views may contain complementary information, but they may also have noise or inconsistencies. Therefore, ensuring structural consistency between different views is crucial for improving clustering performance. Cross-view structural learning is a common technique in multi-view learning, enhancing learning performance by leveraging the consistency between different views. Through cross-view structural learning, the information from multi-views can be effectively merged, thereby improving the generalization ability and robustness of the model. Gcfagg [15] utilizes cross-view feature fusion to achieve consistent expression of multi-view data and explore complementarity between similar samples. UPMGC-S [16] effectively uses the structural information of each view to refine cross-view correspondence. DCMSC [17] integrates diversity and consistency into the data space through constraint learning to realize multi-view clustering. CGL [18] unifies lower-order tensor learning and spectral embedding into a single optimization framework, and on this basis jointly determines spectral embedding matrices and tensor representations. GSF [19] solves the multi-view clustering problem by integrating graph structures of distinct views, which makes good use of the geometric properties of underlying data structures.

Single-cell multi-view clustering is used to analyze single-cell data. In single-cell research, various data views are typically generated, such as gene expression, protein expression, *etc*., with each view providing distinct information. By utilizing these diverse data views to depict the features and structures of single cells more accurately, one can gain a comprehensive understanding of potential patterns and structures within single-cell data, crucially aiding in the in-depth investigation of single-cell omics data. Encluster [20] introduced a multi-view underlying embedding method that generates multiple interpolations for scRNA-seq data using existing imputation techniques to cluster the scRNA-seq data. MCGL [21] achieves more precise clustering results by merging information from distinct views, enhancing the effectiveness of single-cell data analysis. It can effectively manage relationships between different views and comprehensively leverage information from multiple views during the clustering process, thereby improving the accuracy and robustness of clustering. scICML [22] is based on information-theoretic co-clustering that integrates multiple omics single-cell datasets. Through co-clustering, it consolidates the similar features of various data views to reveal cell clustering patterns.

Although existing single-cell multi-view unsupervised clustering methods have made great progress, they still have the following problems: Firstly, due to the various extraction methods for heterogeneous multi-view single-cell data, clustering results heavily rely on the quality of predefined affinity graphs. Secondly, weight matrices of different views and feature weight matrices are often pre-defined, which cannot effectively reflect the importance of distinct views and features, leading to the inability to select useful features and fully consider the inconsistencies between views. Thirdly, many existing multi-view clustering methods utilize graphs as input to reveal the distribution patterns of data, where these graphs are typically pre-processed within each view, overlooking the interconnections of graph structures between multiple views and failing to fully explore underlying relationships among distinct views.

To tackle the challenges in single-cell multi-view clustering, the paper proposes a two-stage multi-view graph spectral clustering method for single-cell RNA-seq data, named MVGSC, to address the aforementioned issues. This two-stage method is based on the ideas of attribute learning and structural learning, combined with the theoretical basis of consistency constraints and complementarity assumptions in multi-view data, to capture complementary and consistent information in multi-view data. The MVGSC framework is illustrated in Fig. (**1**).

The main contributions of the paper include the following three aspects: First, current multi-view clustering algorithms are rarely tailored for single-cell data. The paper concerns single-cell gene expression data and transforms it into linear and non-linear multi-view data through multiple kernel functions processing. Second, the obtained multi-views are treated as two stages: attribute and structure. In the attribute stage, a unified weighted graph U is generated *via* adjusting and merging multi-view similarity graphs, reflecting the importance of distinct views and features while considering inconsistencies among views. The structure stage focuses on the potential relationships between different views, retaining common edges from multi-view similarity graphs to form a unified structure graph A, thus emphasizing the consistency among views. Third, optimization of the attribute and structure graphs was conducted separately by the alternating direction multiplier method. By integrating the weighted graph and structure graph, and applying the graph-based clustering method (spectral clustering) to single-cell data, a comprehensive fusion of multi-view attributes and structural stages is achieved.

## 2 METHODS

### 2.1 Obtain Multi-view Data

Aiming at the problem that a single feature space in scRNA-seq cannot effectively represent cell function, MVGSC comprehensively represents the data of scRNA-seq from distinct perspectives through multi-perspective learning. Due to the high-dimensional, sparse, and noisy characteristics of scRNA-seq data, from a biological perspective, linear and polynomial kernels are suitable for processing high-dimensional, sparse scRNA-seq data. Gaussian kernels have advantages in expanding to large-scale data, and their smoothness helps alleviate the impact of noise in sparse data. Compared with the matrix factorization method NMF based on non-negative constraints, the kernel method obtains more stable multi-view results. Therefore, choosing kernel methods to generate multi-view representations is preferable.

Through multi-kernel function processing, single-cell multi-view data is generated from single-cell data. Specifically, given a set of samples 𝑋, the similarity between cell 𝑥_𝑖_ and cell 𝑥_𝑗_ is calculated.

The first view 𝑉^(1)^ employs the Polynomial kernel function and is computed as Eq. (1).


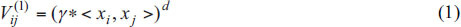


Where 𝛾 used to scale inner products, usually set 𝛾 = 1. and d is the control order.

The second view 𝑉^(2)^ uses the Gaussian kernel function and is computed as Eq. (2).


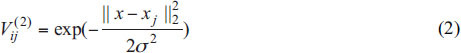


Where σ is a bandwidth parameter.

The third view 𝑉^(3)^ utlizes the linear kernel function and is computed as Eq. (3).


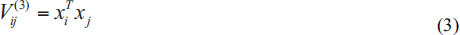


Finally, Multi-view data {𝑉^m^} composed of 𝑉^(1)^ 、 𝑉^(2)^ and 𝑉^(3)^ is obtained.

### 2.2 Multi-view Similarity Graph Construction by KNN

Usually, KNN is used to construct multiple similarity graphs [23, 24]. The similarity connection between samples is determined by calculating the L2 distance. For each sample, KNN will find the K nearest neighbors, which can be considered to have strong connections in the similarity graph. The K value will directly affect the density and strength of the connections in the graph [25]. Initialize matrices {S_v} by using Eq. (4).


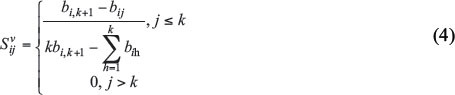


Wherein 
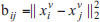
, k stands for neighbours numbers.

### 2.3 Weighted Attributes Graph Construction

By weighting different views to obtain an adaptive weighted graph, it is first necessary to initialize weights of each view and weight it to get attribute graph U. Initialize weight for each view, 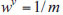. Initialize U *via* connecting {S^v^} with 𝑤 by Eq. (5) .U as average similarity matrix of {S^v^}.


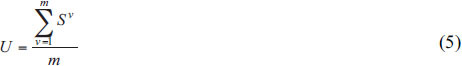


And then F_1_ is obtained by solving Eq. (6).


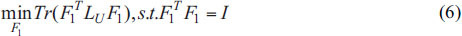


Optimization Muti-view attribute graph are as follows. Firstly, Fix, 𝑤, U, F_1_, update {S^v^} *via* Eq. (7).


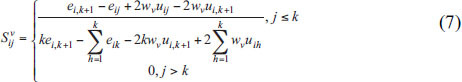


Where 
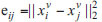
.

Secondly, Fix {S^v^},F_1_ and U, update w by using Eq. (8):


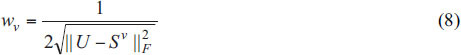


Thirdly, Fix, 𝑤, {S^v^}, F_1_, update U *via* solving problem Eq. (9):


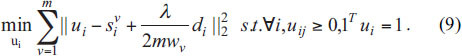


Finally, adaptive weighted graph U as Attribute graph is obtained.

### 2.4 Consistency Structure Graph Construction

Constructing a consistent graph structure between multiple views can offer a deeper understanding of the same underlying structure among multiple views, and better explore potential correlation relationships between views, which is crucial for multi-view clustering [26, 27]. Especially, it preserves consistent information between views using Hadamard products. Generate multi-view structure graph H by using Eq. (10).


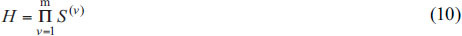


Similarity matrix A will be learned based on H as follows. A is corresponding to the learned intrinsic structure graph. The objective function is as Eq. (11).


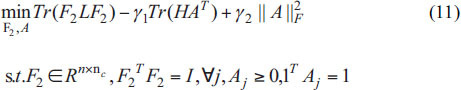


Where 𝛾_1_ and 𝛾_2_ are weight factors. The Eq. (11) can be solved by dividing it into the following two sub problems.

The optimal F_2_ is formed *via* arranging eigenvectors of the first c smallest eigenvalues of Laplacian matrix of A. Finally, the optimized structural graph A is obtained.

### 2.5 Muti-graph Fusion Clustering

#### 2.5.1 Fusion Adaptive Weighted Graph U and Structure Graph A

Fusion Adaptive weighted graph U and Structure graph A using Eq.(12). And construct Symmetric normalized matrix T by using 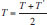.


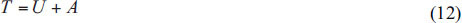


#### 2.5.2 Spectral Clustering

Obtaining the low-dimensional embedding representation of the graph through spectral decomposition, these embeddings are clustered using the k-means algorithm, thus achieving spectral clustering [28-30]. Specifically, the steps are as follows:

Step 1: Construct degree matrix D

For T, the degree matrix D is defined as the sum of weights of adjacent edges of a vertex. Since T is symmetric, the sum of each row can be directly calculated to obtain the degree. To avoid division by zero, a small constant epsilon (eps) is added. The calculation formula for the degree matrix is given in Eq. (13).


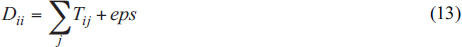


Step 2: Construction Normalized Laplacian Matrix L

Construction Normalized Laplacian Matrix L using Eq.(14).


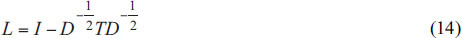


Step 3: Feature decomposition

The eigendecomposition of the normalized Laplacian matrix L is carried out to obtain the eigenvector matrix V using Eq. (15). Here, only the last c eigenvectors are taken, as they typically contain the main structural information of the graph.


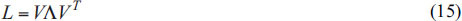


Where V is the eigenvector matrix, and its columns are the eigenvectors f of L. Λ is the eigenvalue diagonal matrix, with its diagonal elements being the eigenvalues of L.

Step 4: Normalization of eigenvectors

To obtain normalized eigenvectors F, normalize the extracted eigenvectors F row-wise using Eq. (16).


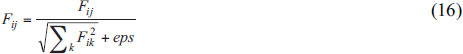


Step 5: Clustering

Finally, applying k-means to cluster normalized eigenvectors F, resulting in c clusters. The MVGSC algorithm is shown in Algorithm 1.


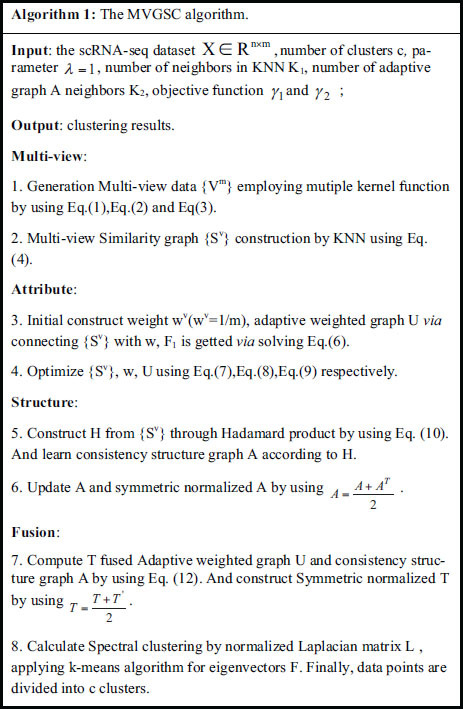


## 3 RESULTS

In this section, these experiments use eight real single-cell datasets to evaluate the performance of the MVGSC model, which uses the MATLAB 2022 development environment. Baseline methods are implemented in Python 3.8 and MATLAB 2022.

### 3.1 The scRNA-seq Data

This study utilized eight real single-cell datasets, namely Usoskin [31], Engel [32], Pollen [33], Darmanis [34], Grover [35], Goolam [36], Zeisel [37], and Yan [38]. Table (**1**) presents the information on single-cell datasets, including their sources, number of cells, number of clusters, the number of genes, and the corresponding species.

### 3.2 Evaluation Metrix

In this study, six clustering metrics are utilized to evaluate the performance of MVGSC. The higher the values of clustering metrics, the better the clustering performance. The Adjusted Rand Index (ARI) value is evaluated and calculated using Eq. (17).


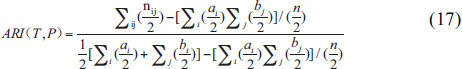


The ARI value ranges between -1 and 1. The closer the ARI value is to 1, the more similar the clustering results are [39]. When the ARI value approaches 0, it indicates that the clustering results are random. A negative value indicates that the difference in clustering results is greater than randomness [40]. The Normalized Mutual Information (NMI) value is evaluated and calculated using Eq. (18).


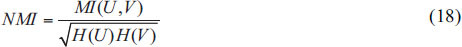



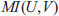
represents the mutual information about true label P and predicted label Q. In addition, H(P) and H(Q) represent the entropy of P and Q, respectively [41, 42]. Evaluate and calculate F-score value using Eq. (19).


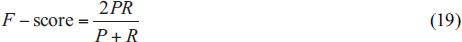


Precision (P) is proportion of positive samples correctly predicted as positive, and Recall (R) is the proportion of all positive samples correctly predicted as positive [43]. Evaluate and calculate Accuracy (ACC) value using Eq. (20).


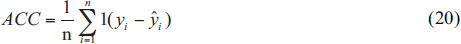


n represents the number of data points, 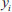 represents true labels, 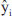 is predicted labels [44, 45]. When the predicted result is consistent with the reality, the accuracy value is 1 [46]. Evaluate and calculate Purity value using Eq. (21).


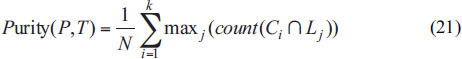


N is the number of samples, 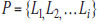 is clusters obtained after clustering, 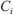 is all samples in the i-th cluster, 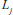 represents true samples in the j-th category, count 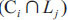 is the number of samples in cluster that belong to 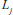 the category [47, 48]. Evaluate and calculate Precision value using Eq. (22).


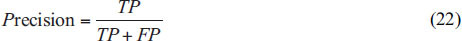


Where True Positive (TP) indicates data of the same type that is correctly classified into the same category. True Negative (TN) refers to data of different types that is incorrectly classified into distinct categories. False Positive (FP) means classifying two data points of different types into the same category. False Negative (FN) refers to data of two different types being classified into different categories [49, 50].

### 3.3 Experimental Results

To further evaluate the clustering performance of MVGSC, it was compared it with 9 competing methods, including traditional single-cell single-view clustering and multi-view clustering methods. Among them, there are T-SNE [51] PCA [52] K-means [5] NMF [53] single-cell single-view clustering methods and GMC [9] GSF [22] FastMICE [54] MVCC [55] MVGL [56] multi-view clustering methods. Among them, the baseline method adopts the default parameters provided by the respective authors. Specifically, Parameter 𝜆 = 1 in GMC. Parameter 𝛾_1_ = 1 and 𝛾_2_ = 1 in GSF, There are two parameters for FastMICE 𝑀 = 20 and 𝑃 = 1000. Parameter 𝛽 = 0.6 in MVCC, There are two parameters for MVGL 𝛽 = 1 and 𝛾 = 1. In MVGSC, set polynomial kernel control parameters d = 2, gaussian kernel bandwidth parameter 𝜎 = 10, objective function initial parameters 𝛾_1_ = 1 and 𝛾_2_ = 1.

In experiment, the MVGSC parameter 𝛾 is set to 1 as its initial value and automatically adjusts. Specifically, in each iteration, if the number of connected components is less than or greater than the number of clusters c, increase (𝛾 = 𝛾 * 2) or decrease (𝛾 = 𝛾 ÷ 2) until the target value is exactly met or very close to the target value, and stop the iteration. The final iteration count for all datasets is less than 20 times. From Table (**2** to **5**) presents ARI、NMI、F1-score and ACC result for all methods.

It can be seen that MVGSC has the highest clustering results on most scRNA-seq datasets. The clustering results of the MVGSC method on the Pollen dataset are lower than those of the MVCC method. This is because the Pollen dataset has low sparsity and an obvious feature distribution, so feature extraction of the data is not complicated, and the differences between multiple views are not obvious. The two-stage advantages of the MVGSC method are not well reflected in the Pollen dataset. In addition, the ARI, NMI, F1-score, and ACC results of the MVGSC method on the Pollen dataset are all above 0.8, indicating that the clustering performance of the MVGSC method on the Pollen dataset is strong.

#### 3.3.1 Single View Clustering Comparision

MVGSC was run on all 8 single-cell datasets and compared with four other popular single-view clustering algorithms, as shown in Fig. (**2**). Analysis experiments show that MVGSC always outperforms other single-cell clustering methods on seven single-cell datasets, but is slightly lower than other methods on the Pollen dataset. Besides, MVGSC performs well on the Usoskin, Pollen, Goolam, and Yan datasets with results above 0.8.

To contrast the performance of MVGSC with other popular multi-view clustering methods, as shown in Fig. (**3**), experiments were conducted on eight different scales of single-cell datasets. MVGSC outperforms other multi-view clustering methods significantly in seven single-cell datasets, with slightly lower performance on the Pollen dataset compared to the MVCC method. Our method achieves values around 0.8 in ARI, NMI, F1-score, ACC, Purity, Precision, and six other evaluation metrics, and performs best on the Usoskin, Goolam, and Yan datasets.

#### 3.3.2 Multi-view Spectral Clustering Analysis

In order to further analyze the key role of multi-kernel learning in multi-view clustering, experiments were conducted on eight single-cell datasets, and the comparison results are shown in Fig. (**4**). The comparison methods include:

(1) *SC*(1): Spectral clustering algorithm from the first perspective.

(2) *SC*(2): Spectral clustering algorithm from the second perspective.

(3) *SC*(3): Spectral clustering algorithm from the third perspective.

(4) *SC*(4): Spectral clustering algorithm.

(5) *MVGSC*(5): The paper method.

In the comparative analysis of the three views of spectral clustering, the performance of the third view of spectral clustering on most datasets is better than that of the first and second views, resulting in higher clustering accuracy, even outperforming the single-view spectral clustering results. This suggests that linear kernels may be more suitable for clustering single-cell data.

#### 3.3.3 Ablation Experiment

In the MVGSC method, multi-view attribute and structure learning are important components of the framework. This section analyzes the impact of the attribute and structural modules on clustering results, and verifies them through ablation experiments. Two variants have been introduced:

(1) w/o structure: MVGSC without structural module.

(2) w/o attribute: MVGCC without attribute module.

**Fig. (5):**
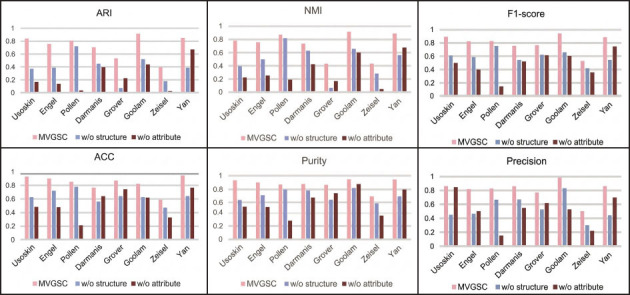
Ablation results. Visualization and data analysis conducted by the authors.

To explore the impact of two variables on the experimental results of the model while keeping other conditions constant, we conducted ablation experiments on eight real single-cell datasets and compared the MVGSC model with the w/o structure model and w/o attribute model. Ablation experiments were performed using all evaluation metrics, and the results are compared as shown in Fig. (**5**). In Fig. **5**, Purity and Precision clustering evaluation metrics were used for quantitative evaluation, and the comparison results are shown in Table **6** and **7**.

#### 3.3.4 Convergence Analysis

The convergence curves of the MVGSC method on four scRNA-seq datasets are shown in Fig. (**6**). The x-axis and y-axis represent the number of iterations and the objective function value, respectively. It can be seen that the target value is inversely proportional to the number of iterations, and the target value reaches its minimum within 10 iterations because we provide optimized solutions for each subproblem. This indicates that the MVGSC method has fully converged, further proving the robustness of the MVGSC method.

#### 3.3.5 Parameter Sensitivity Analysis

Sensitivity analysis of parameters is crucial in clustering algorithms [57, 58]. Parameter insensitivity refers to the experimental results of modifying some parameters of the algorithm should not differ significantly or remain unchanged [59, 60]. The parameters in this paper include 𝛾_1_ and 𝛾_2_. Here, parameter tuning experiments were conducted on 𝛾_1_ and 𝛾_2_, setting the ranges of {10^-3^ -10^1^}. The experimental results on eight datasets are shown in Fig. (**7**). The results manifest that the parameters are sensitive on the Goolam dataset after changing, while the accuracy (ACC) remains unchanged or changes very little in most single-cell datasets, demonstrating the good parameter insensitivity of the MVGSC model.

#### 3.3.6 Time Complexity Analysis

In this section, the time complexity of the MVGSC method proposed by the research institute is discussed. Use the symbol O to describe the computational complexity, assuming n samples, m genes, v views, k neighbors, and N iterations. The time complexity of the MVGSC method is mainly divided into multiple stages. The time complexity for constructing multiple views is O(n^2^m), the time complexity for weighted calculation and optimization of multi-view attribute graphs is O(N(vn^2^k+n^3^)), the time complexity for calculating and optimizing multi-view structural graphs is O(n^3^+vn^2^), the time complexity for fusing multiple graphs is O(n^2^), and the time complexity for spectral clustering is O(n^3^). Due to the small number of iterations N, views v, and neighbors k in all datasets, the overall time complexity of the MVGSC method is O(n^3^).

In addition, we found that ENU [61] and kNN-PL [62] have significant improvements in constructing cell maps compared to traditional K-nearest neighbor methods. Therefore, improving KNN methods may further optimize the construction of better cell maps in the future, and there is hope that this will reduce time complexity.

#### 3.3.7 UMAP Visualization Analysis

In order to intuitively understand the spatial distribution of cell clustering features, Uniform Manifold Approximation and Projection (UMAP) was used to display the distribution of different clusters of cells, as shown in Fig. (**8**). The same color represents the same cell type, while different colors represent different cell clusters. From Fig. (**8**), it can be observed that the MVGSC method can significantly cluster cells of the same class and separate cells of different classes. The results indicate that the clustering model of MVGSC can capture potential hidden information about cells and fully utilize the topological relationships between cells to accurately predict cell clusters.

#### 3.3.8 Gene Enrichment Analysis

**Fig. (9):**
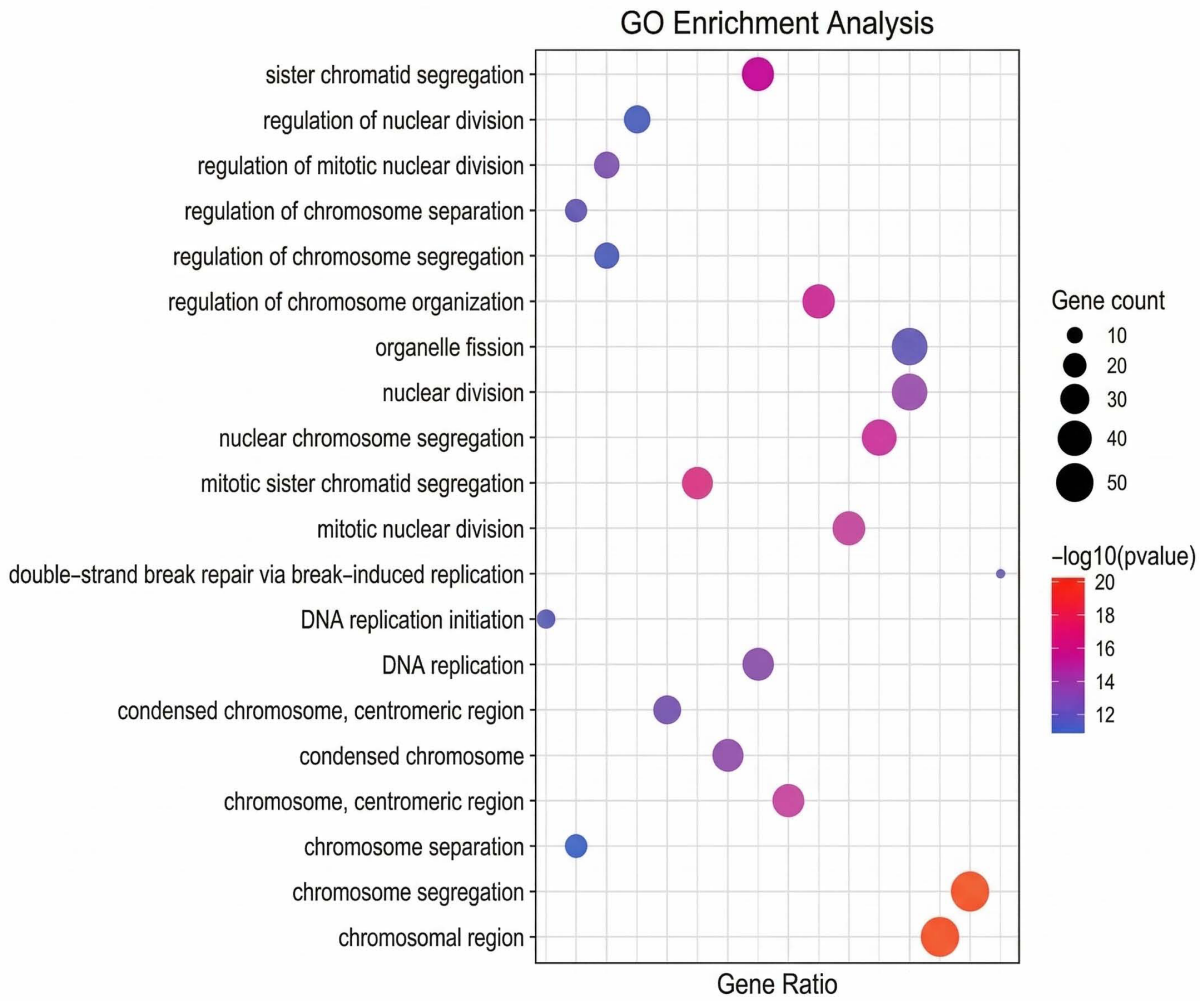
Identify the top twenty GO annotations significantly enriched in clustering. Visualization and data analysis conducted by the authors.

**Fig. (10):**
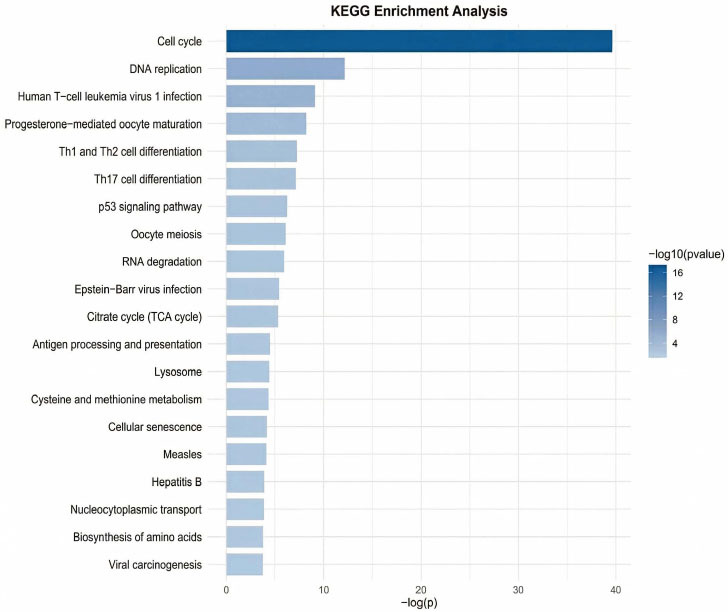
Identifying the top twenty KEGG pathways significantly enriched. Visualization and data analysis conducted by the authors.

By analyzing the feature space distribution of the Engel dataset in Fig. (**8**), most cells of cell type 2 and cell type 4 exhibit significant clustering in the reduced dimensional space. To explore the biological driving factors of clustering, this section conducts gene co-expression analysis experiments. Gene Ontology (GO) enrichment analysis and Kyoto Encyclopedia of Genes and Genomes (KEGG) pathway enrichment analysis are shown in Figs. **9** and **10**, respectively.

Based on the results of spatial clustering and GO functional enrichment, the differentially expressed genes between cell type 2 and cell type 4 were significantly enriched in cell cycle regulation, chromosome segregation, and DNA replication-related functions. It is inferred that the two represent subpopulations of cells at different stages of proliferation or with different proliferative activities. The difference in proliferation status and cell cycle-related gene expression between the two types of cells drives their significant separation in the dimensionality reduction space.

From the KEGG pathway enrichment analysis results, it can be seen that there are more differentially expressed genes between cell type 2 and cell type 4 on the cell cycle pathway “Cell cycle.” Therefore, it can be inferred that the two may be the same type of cells at different cell cycle stages, resulting in differences in proliferation status due to their different stages of the cell cycle. In summary, by validating clusters based on known cell types and biological processes, the gap between computational performance and biological significance can be bridged.

## 4 DISCUSSION

The advantages of two-stage multi-view learning for MVGSC are as follows: firstly, the basic features of the problem are clarified through attribute learning, and then the underlying correlation relationships between views are established through structural learning. Dual-stage multi-view learning can more accurately capture complex relationships in the data, thereby improving the accuracy of the model. Secondly, two-stage multi-view learning can better capture consistency and complementary information in multi-view data, thereby enhancing the model's generalization ability. Additionally, two-stage multi-view learning allows for independent attribute and structure learning for each view, enabling more flexible handling of differences and complexities between different views. When the features of scRNA-seq data are complex and there are significant differences between views, the two-stage method can better capture the complex relationships in the data, demonstrating superior performance compared to a single framework. However, the two-stage method also has certain limitations, such as an increase in computational complexity. In the future, cell graph construction can use improved methods to further reduce time complexity and optimize multi-view clustering.

## CONCLUSION

In the context of multi-view clustering, this paper proposes a two-stage multi-view graph spectral clustering method for single-cell RNA-seq data (MVGSC), which transforms single-cell data analysis into multi-perspective analysis by jointly exploring the inconsistency and consistency between multiple views in the attribute and structure stages.

As current multi-view clustering algorithms are rarely designed for scRNA-seq data, the paper focuses on single-cell gene expression data, transforming it into multi-view data with both linear and nonlinear characteristics through multi-kernel function processing. The attribute stage involves adjusting weights and integrating multiple perspective similarity graphs to get a unified weighted graph, reflecting the importance of different views and features while fully considering the inconsistency among views. The structure stage focuses on exploring the potential relationships between different views, preserving common edges from multiple perspective similarity graphs to form a unified structural graph, emphasizing the consistency among views. Clustering of scRNA-seq data is performed by integrating the weighted graph and structural graph, taking into account both the inconsistency among views and the consistency between views. Clustering results are obtained by applying graph clustering methods (spectral clustering). Compared with several state-of-the-art clustering methods, MVGSC achieves more satisfactory results on eight commonly used single-cell datasets, demonstrating through analysis the effectiveness and robustness of the proposed MVGSC.

## Figures and Tables

**Fig. (1):**
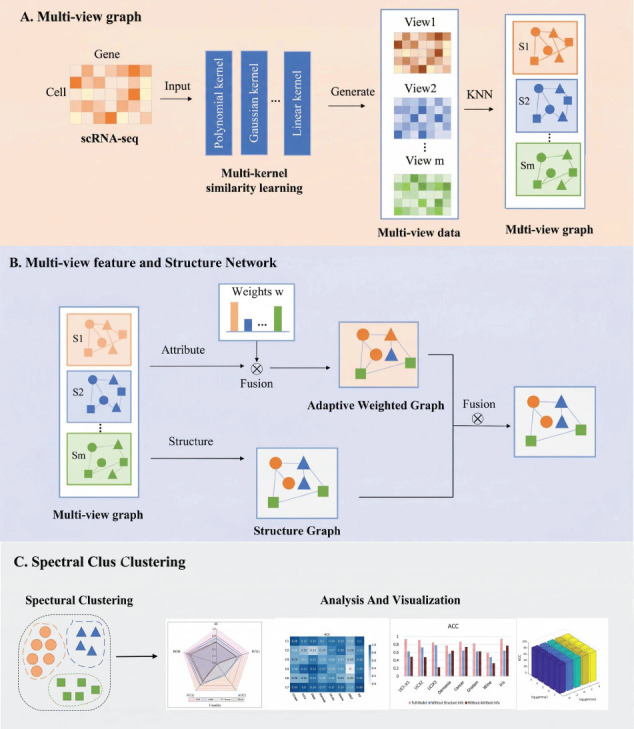
MVGSC framework. Original figure created by the authors.

**Fig. (2):**
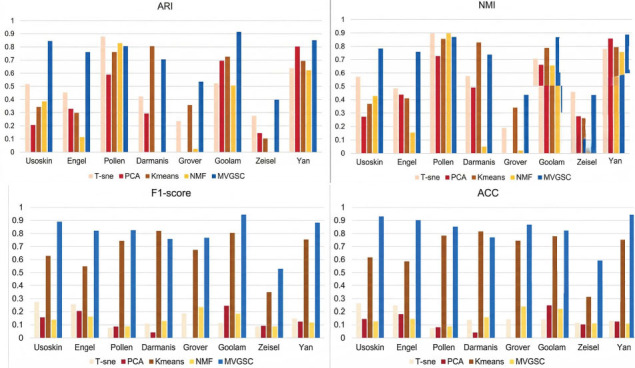
Comparison with single-cell clustering method. Visualization and data analysis conducted by the authors.

**Fig. (3):**
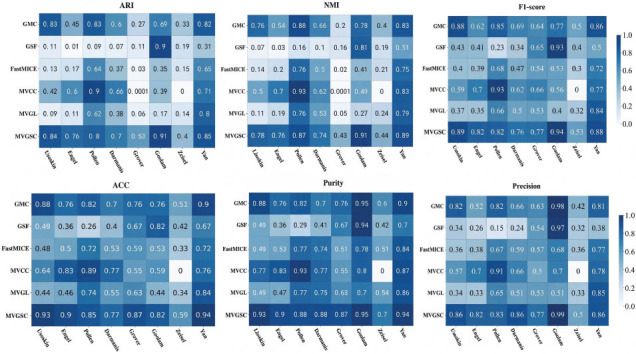
Compares with the multi-view clustering method. Note: 0 indicates incorrect implementation. Visualization and data analysis conducted by the authors.

**Fig. (4):**
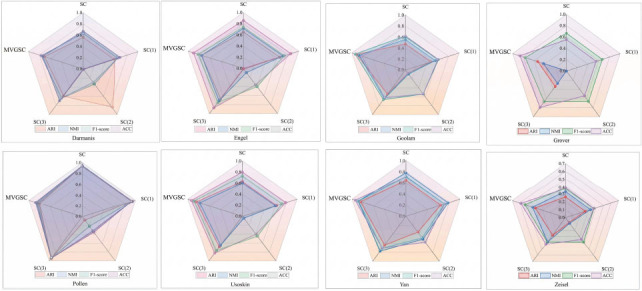
Comparison of multiple similarity views of spectral clustering. Visualization and data analysis conducted by the authors.

**Fig. (6):**
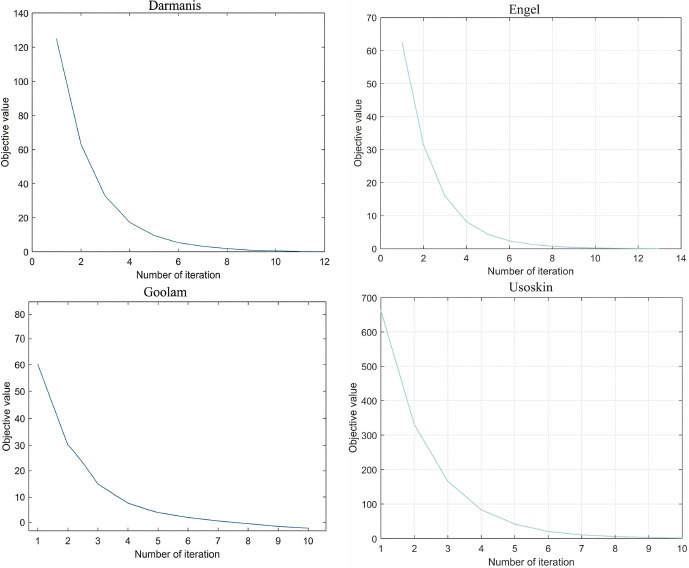
Convergence behaviors of MVGSC. Visualization and data analysis conducted by the authors.

**Fig. (7):**
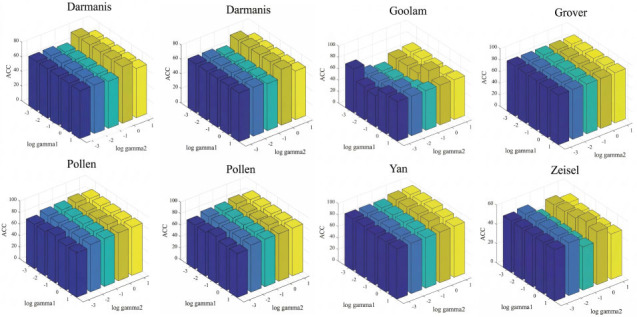
Parameter 𝛾_1_ ands 𝛾_2_ ensitivity analysis. Visualization and data analysis conducted by the authors.

**Fig. (8):**
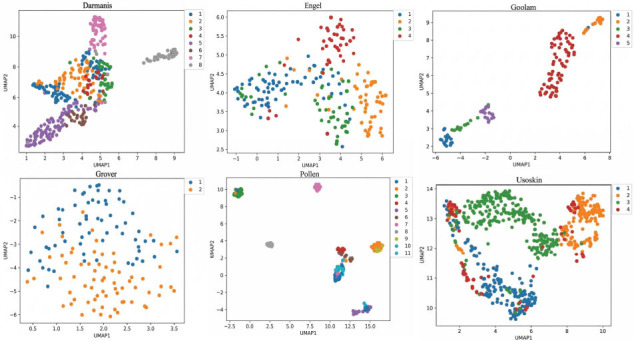
UMAP clustering visualization. Visualization and data analysis conducted by the authors.

**Table 1:** The information of single-cell datasets.

**Dataset**	**Source**	**Cells**	**Types**	**Genes**	**Species**
Usoskin	GSE59739	622	4	17772	Mus musculus
Engel	GSE74597	203	4	23337	Homo sapiens
Pollen	SRP041736	249	11	14805	Homo sapiens
Darmanis	GSE67835	420	8	22085	Homo sapiens
Grover	GSE70657	135	2	147	Mus musculus
Goolam	E-MTAB-3321	124	5	40315	Mus musculus
Zeisel	GSE60361	3005	9	4112	Mus musculus
Yan	GSE36552	90	6	19594	Human preimplantation

**Table 2:** ARI result for all methods.

**ACC**	**Usoskin**	**Engel**	**Pollen**	**Darmanis**	**Grover**	**Goolam**	**Zeisel**	**Yan**
t-sne	0.51	0.45	**0.87**	0.42	0.23	0.52	0.27	0.63
pca	0.20	0.32	0.58	0.29	-	0.69	0.14	0.80
kmeans	0.34	0.29	0.76	0.80	0.35	0.72	0.10	0.69
NMF	0.38	0.11	0.82	0.01	0.02	0.50	-	0.61
GMC	0.83	0.45	0.83	0.6	0.27	0.69	0.33	0.82
GSF	0.11	0.01	0.09	0.07	0.11	0.9	0.19	0.31
FastMICE	0.13	0.17	0.64	0.37	0.03	0.35	0.15	0.65
MVCC	0.42	0.6	0.9	0.66	-	0.39	-	0.71
MVGL	0.09	0.11	0.62	0.38	0.06	0.17	0.14	0.8
MVGSC	**0.84**	**0.75**	0.84	**0.70**	**0.53**	**0.91**	**0.39**	**0.85**

**Table 3:** NMI result for all methods.

**NMI**	**Usoskin**	**Engel**	**Pollen**	**Darmanis**	**Grover**	**Goolam**	**Zeisel**	**Yan**
t-sne	0.57	0.48	0.90	0.57	0.18	0.70	**0.45**	0.78
pca	0.27	0.43	0.72	0.48	-	0.65	0.27	0.85
kmeans	0.36	0.41	0.85	**0.82**	0.33	0.78	0.26	0.79
NMF	0.42	0.15	0.89	0.04	0.02	0.65	0.01	0.75
GMC	0.76	0.54	0.88	0.66	0.2	0.78	0.4	0.83
GSF	0.07	0.03	0.16	0.1	0.16	0.81	0.19	0.51
FastMICE	0.14	0.2	0.76	0.5	0.02	0.41	0.21	0.75
MVCC	0.5	0.7	**0.93**	0.62	-	0.49	-	0.83
MVGL	0.11	0.19	0.76	0.53	0.05	0.27	0.24	0.79
MVGSC	**0.78**	**0.75**	0.86	0.73	**0.43**	**0.86**	0.43	**0.88**

**Table 4:** F1-score result for all methods.

**F1-score**	**Usoskin**	**Engel**	**Pollen**	**Darmanis**	**Grover**	**Goolam**	**Zeisel**	**Yan**
t-sne	0.27	0.25	0.07	0.10	0.18	0.11	0.08	0.14
pca	0.15	0.20	0.83	0.04	-	0.24	0.09	0.12
kmeans	0.62	0.54	0.74	**0.81**	0.67	0.80	0.34	0.75
NMF	0.13	0.16	0.08	0.12	0.23	0.18	0.08	0.11
GMC	**0.88**	0.62	0.85	0.69	0.64	0.77	0.5	0.86
GSF	0.43	0.41	0.23	0.34	0.65	0.93	0.4	0.5
FastMICE	0.4	0.39	0.68	0.47	0.54	0.53	0.3	0.72
MVCC	0.59	0.70	**0.93**	0.62	0.66	0.56	-	0.77
MVGL	0.37	0.35	0.66	0.5	0.53	0.4	0.32	0.84
MVGSC	**0.88**	**0.82**	0.82	0.75	**0.76**	**0.94**	**0.52**	**0.88**

**Table 5:** ACC result for all methods.

**ACC**	**Usoskin**	**Engel**	**Pollen**	**Darmanis**	**Grover**	**Goolam**	**Zeisel**	**Yan**
t-sne	0.26	0.24	0.07	0.14	0.17	0.14	0.11	0.13
pca	0.14	0.18	0.07	0.04	0.01	0.24	0.10	0.12
kmeans	0.61	0.58	0.78	**0.81**	0.74	0.78	0.31	0.74
NMF	0.12	0.14	0.08	0.15	0.23	0.21	0.11	0.1
GMC	0.88	0.76	0.82	0.7	0.76	0.76	0.51	0.9
GSF	0.49	0.36	0.26	0.4	0.67	0.67	0.42	0.67
FastMICE	0.48	0.5	0.72	0.53	0.59	0.59	0.33	0.72
MVCC	0.64	0.83	**0.89**	0.77	0.55	0.55	-	0.76
MVGL	0.44	0.46	0.74	0.55	0.63	0.63	0.34	0.84
MVGSC	**0.93**	**0.90**	0.85	0.76	**0.86**	**0.86**	**0.58**	**0.94**

**Table 6:** Purity ablation experiment result for MVGSC.

**Purity**	**w/o Structure**	**w/o Attribute**	**MVGSC**
Usoskin	0.6398	0.5289	**0.9308**
Engel	0.7192	0.5221	**0.9014**
Pollen	0.8032	0.2771	**0.8755**
Darmanis	0.7880	0.6738	**0.8833**
Grover	0.6444	0.7407	**0.8666**
Goolam	0.8225	0.879	**0.9516**
Zeisel	0.5830	0.3507	**0.6995**
Yan	0.7	0.8	**0.9444**

**Table 7:** Precision ablation experiment result for MVGSC.

**Precision**	**w/o Structure**	**w/o Attribute**	**MVGSC**
Usoskin	0.4511	0.8464	**0.8609**
Engel	0.4652	0.5010	**0.8202**
Pollen	0.6677	0.1518	**0.8290**
Darmanis	0.6680	0.5462	**0.8579**
Grover	0.5274	0.6153	**0.7697**
Goolam	0.8319	0.5289	**0.9852**
Zeisel	0.3003	0.2190	**0.5016**
Yan	0.4446	0.6953	**0.8600**

## Data Availability

The data that supports the findings of this study are available from the corresponding author [JS], upon reasonable request.

## LIST OF ABBREVIATIONS

ACC: Accuracy
ARI: Adjusted Rand Index
FN: False Negative
FP: False Positive
GO: Gene Ontology
KEGG: Kyoto Encyclopedia of Genes and Genomes
KNN: K Nearest Neighbors
MKL: Multiple Kernel Learning
NMI: Normalized Mutual Information
scRNA-seq: single-cell RNA sequencing
TP: True Positive
UMAP: Uniform Manifold Approximation and Projection

## References

[1] Wu W., Ma X. (2020). Joint learning dimension reduction and clustering of single-cell RNA-sequencing data.. Bioinformatics.

[2] Wan H., Chen L., Deng M. (2022). scNAME: neighborhood contrastive clustering with ancillary mask estimation for scRNA-seq data.. Bioinformatics.

[3] Wang Y., Wong K.C., Li X. (2022). Exploring high-throughput biomolecular data with multiobjective robust continuous clustering.. Inf. Sci..

[4] Yu L., Cao Y., Yang J.Y.H., Yang P. (2022). Benchmarking clustering algorithms on estimating the number of cell types from single-cell RNA-sequencing data.. Genome Biol..

[5] Grün D., Lyubimova A., Kester L., Wiebrands K., Basak O., Sasaki N., Clevers H., van Oudenaarden A. (2015). Single-cell messenger RNA sequencing reveals rare intestinal cell types.. Nature.

[6] Zheng R., Li M., Liang Z., Wu F.X., Pan Y., Wang J. (2019). SinNLRR: a robust subspace clustering method for cell type detection by non-negative and low-rank representation.. Bioinformatics.

[7] Wang B., Zhu J., Pierson E., Ramazzotti D., Batzoglou S. (2017). Visualization and analysis of single-cell RNA-seq data by kernel-based similarity learning.. Nat. Methods.

[8] Ng A., Jordan M., Weiss Y. (2001). On spectral clustering: Analysis and an algorithm.. Adv. Neural Inf Process. Syst.

[9] Wang H., Yang Y., Liu B. (2020). GMC: Graph-based multi-view clustering.. IEEE Trans. Knowl. Data Eng..

[10] Peng X., Huang Z., Lv J., Zhu H., Zhou J.T. (2019). COMIC: Multi-view clustering without parameter selection.. Proceedings of the 36th International Conference on Machine Learning (ICML).

[11] Chen M.S., Huang L., Wang C.D., Huang D. (2020). Multi-view clustering in latent embedding space.. Proc. Conf. AAAI Artif. Intell..

[12] Lin Y., Zou G. (2021). Clustering single-cell RNA sequencing data by multi-view latent embedding learning.. Proceedings of the 2021 International Conference on Computer Information Science and Artificial Intelligence (CISAI).

[13] Wu W., Zhang W., Hou W., Ma X. (2023). Multi-view clustering with graph learning for scRNA-seq data.. IEEE/ACM Trans. Comput. Biol. Bioinformatics.

[14] Zeng P., Lin Z. (2024). scICML: Information-theoretic co-clustering-based multi-view learning for the integrative analysis of single-cell multi-omics data.. IEEE/ACM Trans. Comput. Biol. Bioinformatics.

[15] Qi R., Wu J., Guo F., Xu L., Zou Q. (2021). A spectral clustering with self-weighted multiple kernel learning method for single-cell RNA-seq data.. Brief. Bioinform..

[16] Liu B.Y., Huang L., Wang C.D., Fan S., Yu P.S. (2021). Adaptively weighted multiview proximity learning for clustering.. IEEE Trans. Cybern..

[17] (2023). Adaptive learning-based multi-view unsupervised feature selection method.. Jisuanji Yingyong.

[18] Yan W., Zhang Y., Lv C., Tang C., Yue G., Liao L., Lin W. (2023). GCFAgg: Global and cross-view feature aggregation for multi-view clustering.. Proceedings of the IEEE/CVF Conference on Computer Vision and Pattern Recognition (CVPR).

[19] Wang Y., Chang D., Fu Z., Wen J., Zhao Y. (2023). Incomplete multiview clustering via cross-view relation transfer.. IEEE Trans. Circ. Syst. Video Tech..

[20] Li Z., Tang C., Chen J., Wan C., Yan W., Liu X. (2019). Diversity and consistency learning guided spectral embedding for multi-view clustering.. Neurocomputing.

[21] Li Z., Tang C., Liu X., Zheng X., Zhang W., Zhu E. (2022). Consensus graph learning for multi-view clustering.. IEEE Trans. Multimed..

[22] Zhan K., Niu C., Chen C., Nie F., Zhang C., Yang Y. (2019). Graph structure fusion for multiview clustering.. IEEE Trans. Knowl. Data Eng..

[23] Steinbach M., Tan P.N. (2009). kNN: k-nearest neighbors.. The Top Ten Algorithms in Data Mining..

[24] Zhang S., Li X., Zong M., Zhu X., Wang R. (2018). Efficient kNN classification with different numbers of nearest neighbors.. IEEE Trans. Neural Netw. Learn. Syst..

[25] Zhang H., Kiranyaz S., Gabbouj M. (2018). Data clustering based on community structure in mutual k-nearest neighbor graph. *41st International Conference on Telecommunications and Signal. Processing (TSP),* Athens, Greece.

[26] Fu L., Lin P., Vasilakos A.V., Wang S. (2020). An overview of recent multi-view clustering.. Neurocomputing.

[27] Fang U., Li M., Li J., Gao L., Jia T., Zhang Y. (2023). A comprehensive survey on multi-view clustering.. IEEE Trans. Knowl. Data Eng..

[28] Tang C., Li Z., Wang J., Liu X., Zhang W., Zhu E. (2023). Unified one-step multi-view spectral clustering.. IEEE Trans. Knowl. Data Eng..

[29] Wu J., Lin Z., Zha H. (2019). Essential tensor learning for multi-view spectral clustering.. IEEE Trans. Image Process..

[30] Huang D., Wang C.D., Wu J.S., Lai J-H., Kwoh C-K. (2020). Ultra-scalable spectral clustering and ensemble clustering.. IEEE Trans. Knowl. Data Eng..

[31] Usoskin D., Furlan A., Islam S., Abdo H., Lönnerberg P., Lou D., Hjerling-Leffler J., Haeggström J., Kharchenko O., Kharchenko P.V., Linnarsson S., Ernfors P. (2015). Unbiased classification of sensory neuron types by large-scale single-cell RNA sequencing.. Nat. Neurosci..

[32] Engel I., Seumois G., Chavez L., Samaniego-Castruita D., White B., Chawla A., Mock D., Vijayanand P., Kronenberg M. (2016). Innate-like functions of natural killer T cell subsets result from highly divergent gene programs.. Nat. Immunol..

[33] Pollen A.A., Nowakowski T.J., Shuga J., Wang X., Leyrat A.A., Lui J.H., Li N., Szpankowski L., Fowler B., Chen P., Ramalingam N., Sun G., Thu M., Norris M., Lebofsky R., Toppani D., Kemp D.W., Wong M., Clerkson B., Jones B.N., Wu S., Knutsson L., Alvarado B., Wang J., Weaver L.S., May A.P., Jones R.C., Unger M.A., Kriegstein A.R., West J.A.A. (2014). Low-coverage single-cell mRNA sequencing reveals cellular heterogeneity and activated signaling pathways in developing cerebral cortex.. Nat. Biotechnol..

[34] Darmanis S., Sloan S.A., Zhang Y., Enge M., Caneda C., Shuer L.M., Hayden Gephart M.G., Barres B.A., Quake S.R. (2015). A survey of human brain transcriptome diversity at the single cell level.. Proc. Natl. Acad. Sci. USA.

[35] Grover A., Sanjuan-Pla A., Thongjuea S., Carrelha J., Giustacchini A., Gambardella A., Macaulay I., Mancini E., Luis T.C., Mead A., Jacobsen S.E.W., Nerlov C. (2016). Single-cell RNA sequencing reveals molecular and functional platelet bias of aged haematopoietic stem cells.. Nat. Commun..

[36] Goolam M., Scialdone A., Graham S.J.L., Macaulay I.C., Jedrusik A., Hupalowska A., Voet T., Marioni J.C., Zernicka-Goetz M. (2016). Heterogeneity in Oct4 and Sox2 targets biases cell fate in 4-cell mouse embryos.. Cell.

[37] Zeisel A., Muñoz-Manchado A.B., Codeluppi S., Lönnerberg P., La Manno G., Juréus A., Marques S., Munguba H., He L., Betsholtz C., Rolny C., Castelo-Branco G., Hjerling-Leffler J., Linnarsson S. (2015). Cell types in the mouse cortex and hippocampus revealed by single-cell RNA-seq.. Science.

[38] Yan L., Yang M., Guo H., Yang L., Wu J., Li R., Liu P., Lian Y., Zheng X., Yan J., Huang J., Li M., Wu X., Wen L., Lao K., Li R., Qiao J., Tang F. (2013). Single-cell RNA-Seq profiling of human preimplantation embryos and embryonic stem cells.. Nat. Struct. Mol. Biol..

[39] Yeung K.Y., Ruzzo W.L. (2001). Principal component analysis for clustering gene expression data.. Bioinformatics.

[40] Zhang S., Wong H.S., Shen Y. (2012). Generalized adjusted rand indices for cluster ensembles.. Pattern Recognit..

[41] McDaid A.F., Greene D., Hurley N. (2011). Normalized mutual information to evaluate overlapping community finding algorithms.. arXiv.

[42] Amelio A., Pizzuti C. (2017). Correction for closeness: Adjusting normalized mutual information measure for clustering comparison.. Comput. Intell..

[43] Gómez-Adorno H., Aleman Y., Ayala D.V., Sanchez-Perez M.A. (2017). Author clustering using hierarchical clustering analysis. *Working Notes of CLEF 2017 - Conference and Labs of the Evaluation Forum*.

[44] Song C., Liu F., Huang Y. Auto-encoder based data clustering.. Progress in Pattern Recognition, Image Analysis, Computer Vision, and Applications 18th Iberoamerican Congress, CIARP 2013.

[45] Li H., Li H., Wei K. (2018). Automatic fast double KNN classification algorithm based on ACC and hierarchical clustering for big data.. Int. J. Commun. Syst..

[46] Huang P., Huang Y., Wang W., Wang L. (2014). Deep embedding network for clustering.. 22nd International Conference on Pattern Recognition (ICPR).

[47] Kesuma Dinata R., Retno S., Hasdyna N. (2021). Minimization of the number of iterations in K-medoids clustering with purity algorithm.. Rev. Intell. Artif..

[48] Marutho D., Handaka S.H., Wijaya E. (2018). The determination of cluster number at k-mean using elbow method and purity evaluation on headline news.. 2018 International Seminar on Application for Technology of Information and Communication.

[49] Lingras P., Chen M., Miao D. (2008). Precision of rough set clustering.. rough sets and current trends in computing 6th International Conference,.

[50] Gui-Fen C., Li-Ying C., Guo-Wei W., Bao-Cheng W., Da-You L., Sheng-Sheng W. (2007). Application of a spatial fuzzy clustering algorithm in precision fertilisation.. N. Z. J. Agric. Res..

[51] Van der Maaten L., Hinton G. (2008). Visualizing data using t-SNE.. J. Mach. Learn. Res..

[52] Wold S., Esbensen K., Geladi P. (1987). Principal component analysis.. Chemom. Intell. Lab. Syst..

[53] Lee D.D., Seung H.S. (1999). Learning the parts of objects by non-negative matrix factorization.. Nature.

[54] Huang D., Wang C.D., Lai J.H. (2023). Fast multi-view clustering via ensembles: Towards scalability, superiority, and simplicity.. IEEE Trans. Knowl. Data Eng..

[55] Zhan K., Nie F., Wang J., Yang Y. (2019). Multiview consensus graph clustering.. IEEE Trans. Image Process..

[56] Zhan K., Zhang C., Guan J., Wang J. (2018). Graph learning for multiview clustering.. IEEE Trans. Cybern..

[57] Roux S., Buis S., Lafolie F., Lamboni M. (2021). Cluster-based GSA: Global sensitivity analysis of models with temporal or spatial outputs using clustering.. Environ. Model. Softw..

[58] Wang L., Xu Y.P., Xu J., Gu H., Bai Z., Zhou P., Yu H., Guo Y. (2024). Increasing parameter identifiability through clustered time-varying sensitivity analysis.. Environ. Model. Softw..

[59] Dai Z., Scott M.J. (2007). Product platform design through sensitivity analysis and cluster analysis.. J. Intell. Manuf..

[60] Krzak M., Raykov Y., Boukouvalas A., Cutillo L., Angelini C. (2019). Benchmark and parameter sensitivity analysis of single-cell RNA sequencing clustering methods.. Front. Genet..

[61] Kumar A., Singh D., Yadav R.S. (2024). Entropy and improved k‐nearest neighbor search based under‐sampling (ENU) method to handle class overlap in imbalanced datasets.. Concurr. Comput..

[62] Cottrell S., Hozumi Y., Wei G.W. (2024). K-nearest-neighbors induced topological PCA for single cell RNA-sequence data analysis.. Comput. Biol. Med..

